# Effects of DNA methylation on cardiometabolic risk factors: a systematic review and meta-analysis

**DOI:** 10.1186/s13690-022-00907-1

**Published:** 2022-06-02

**Authors:** Zahra Barouti, Motahar Heidari-Beni, Anahita Shabanian-Boroujeni, Morteza Mohammadzadeh, Vida Pahlevani, Parnian Poursafa, Fatemeh Mohebpour, Roya Kelishadi

**Affiliations:** 1 Department of Pediatrics, Child Growth and Development Research Center, Research Institute for Primordial Prevention of Non-Communicable Disease, Isfahan University of Medical Sciences, Hezar Jerib St, Isfahan, Iran; 2 Department of Nutrition, Child Growth and Development Research Center, Research Institute for Primordial Prevention of Non-Communicable Disease, Isfahan University of Medical Sciences, Isfahan, Iran; 3 Isfahan University of Medical Sciences, Isfahan, Iran; 4 Department of Cellular and Molecular Biology, Faculty of medicine, Isfahan University of Medical Sciences, Isfahan, Iran

**Keywords:** Epigenomics, DNA methylation, Cardiometabolic risk factors, Meta-analysis

## Abstract

**Background:**

Epigenetic changes, especially DNA methylation have a main role in regulating cardiometabolic disorders and their risk factors. This study provides a review of the current evidence on the association between methylation of some genes (LINE1, ABCG1, SREBF1, PHOSPHO1, ADRB3, and LEP) and cardiometabolic risk factors.

**Methods:**

A systematic literature search was conducted in electronic databases including Web of Science, PubMed, EMBASE, Google Scholar and Scopus up to end of 2020. All observational human studies (cross-sectional, case–control, and cohort) were included. Studies that assessed the effect of DNA methylation on cardiometabolic risk factors were selected.

**Results:**

Among 1398 articles, eight studies and twenty-one studies were included in the meta-analysis and the systematic review, respectively. Our study showed ABCG1 and LINE1 methylation were positively associated with blood pressure (Fisher’s zr = 0.07 (0.06, 0.09), 95% CI: 0.05 to 0.08). Methylation in LINE1, ABCG1, SREBF1, PHOSPHO1 and ADRB3 had no significant association with HDL levels (Fisher’s zr = − 0.05 (− 0.13, 0.03), 95% CI:-0.12 to 0.02). Positive association was existed between LINE1, ABCG1 and LEP methylation and LDL levels (Fisher’s zr = 0.13 (0.04, 0.23), 95% CI: 0.03 to 0.23). Moreover, positive association was found between HbA1C and ABCG1 methylation (Fisher’s zr = 0.11 (0.09, 0.13), 95% CI: 0.09 to 0.12). DNA methylation of LINE1, ABCG1 and SREBF1 genes had no significant association with glucose levels (Fisher’s zr = 0.01 (− 0.12, 0.14), 95% CI:-0.12 to 0.14).

**Conclusion:**

This meta-analysis showed that DNA methylation was associated with some cardiometabolic risk factors including LDL-C, HbA1C, and blood pressure.

**Registration:**

Registration ID of the protocol on PROSPERO is CRD42020207677.

## Background

Non-communicable diseases (NCDs) are a growing public health concern worldwide. High blood pressure, dyslipidemia, insulin resistance, and obesity are the major cardiometabolic risk factors associated with NCDs [1–3]. Lifestyle and environmental factors interact with epigenetic and lead to metabolic disorders [4, 5]. Epigenetic is a reversible and dynamic changes in gene expression to mediate the environmental effects on cellular functions. DNA methylation is the main epigenetic modifications without any changes in the DNA sequence. DNA methylation has a key role in the development of cardiometabolic disorders and their risk factors [6]. Several studies have assessed the relationship between DNA methylation at different loci of different genes with cardiometabolic risk factors. However, there are inconsistent findings [7–10]. Some results did not show any significant association between DNA methylation and cardiometabolic risk factors [9, 11], whereas some of them confirmed these associations [12–16].

Study on epigenetic changes and their association with cardio-metabolic disorders can be a valuable way for prevention and treatment of NCDs [5, 17]. Several epigenetic changes might affect cardiometabolic risk factors. Some genes, including Long interspersed nuclear element-1 (LINE1), ATP-binding cassette sub-family G member 1 (ABCG1), Sterol regulatory element-binding transcription factor 1 (SREBF1), Phosphatase orphan 1 (PHOSPHO1), Adrenoceptor Beta 3 (ADRB3) and Leptin (LEP) have been assessed more than others. This study aims to provide a summary of the literature that evaluated the relationship between methylation of different cytosine-phosphate-guanine (CpG) sites of LINE1, ABCG1, SREBF1, PHOSPHO1, ADRB3 and LEP genes and cardiometabolic risk factors including high-density lipoprotein cholesterol (HDL), low-density lipoprotein cholesterol (LDL), Hemoglobin A1C (HbA1C), blood glucose and blood pressure (BP).

## Methods

### Search strategy and study selection

The present study was conducted according to the guideline of Preferred Reporting Items for Systematic Reviews and Meta-Analyses, PRISMA [18]. The protocol was registered on PROSPERO (ID: CRD42020207677). A systematic literature search was conducted in Web of Science, Medical databases (PubMed), EMBASE, Google Scholar and Scopus, up to end of 2020. The following search terms were used: (“Epigenetic OR “Epigenomic” OR “methylation” OR “acetylation”) AND (“Metabolic syndrome” OR “MetS” OR “insulin resistance syndrome” OR “cardiometabolic” OR “cardiometabolic risk factor”). We assessed the list of review articles references to find undetected relevant studies. Two researchers screened the records independently and selected relevant studies. In cases of nonconformity, the senior researchers were consulted. Titles and abstracts of the records were checked based on the inclusion and exclusion criteria.

### Inclusion and exclusion criteria

Studies with the following criteria were included: 1. All observational studies (cross-sectional, case–control, and cohort) on adult individuals; 2. English language studies; 3. Studies that assessed the effect of epigenetic changes on cardiometabolic risk factors including HDL, LDL, Total cholesterol, glucose (FBS (fasting blood sugar) and random glucose levels), insulin levels, homeostatic model assessment for insulin resistance (HOMA-IR) and Hb1AC, without restriction of gender, race, ethnicity and year of publication; 4. Studies with available and extractable data; 5.Studies that extracted DNA from blood or tissue.

Papers with the following criteria were excluded: 1. Animal studies 2. Duplicate publications; 3. RNA epigenetic changes.

### Data extraction

After screening studies, the number of articles on some genes including LINE-1, ABCG1, PHOSPHO1, SREBF1, LPL and ADRB3 were more than others; therefore, meta-analyses were particularly conducted on these six genes. The following information was extracted from eligible studies: bibliographic details (first author, publication year, and study design), sample size, participant’s characteristics (gender, age, and body mass index), type of tissue sample, covariate variables, genes and loci, and genes methylation associated with HDL, LDL, glucose, blood pressure, and Hb1AC.

### Quality assessment

We assessed the quality of studies using the Strengthening the Reporting of Observational studies in Epidemiology guidelines (STROBE) checklist [19, 20]. This checklist consists of 22 items with different sections including title and abstract (1 item), introduction (2 items), methods (9 items), results (5 items), sections (4 items), and other information (1 item). Range of score is between zero and twenty-two. Two reviewers assessed the quality of articles independently, and in case of disagreement between the assigned scores, consulted with the third reviewer.

### Statistical analysis

The Correlation coefficient of selected studies was applied for pooled analysis. The potential heterogeneity across studies was evaluated using the Cochran’s Q-test and expressed using the I^2^ index. The pooled results were calculated by the random-effects model. Subgroup analyses based on CpG sites were performed to seek the sources of heterogeneity. In addition, meta-regression was used for assessing the mean age, mean BMI, sample size, and the year of publication of studies as the possible source of heterogeneity. The sensitivity of analysis was performed by excluding one study at a time to gauge the robustness of our results. Publication bias was evaluated by Funnel plot and Egger’s test. The possible publication’s bias was adjusted using the trim and fill method. All statistical analyses were conducted using the STATA 12.0 software (STATA Corp, College Station, Texas, USA). The significance level was set at *p* < 0.05.

## Results

**Fig. 1:**
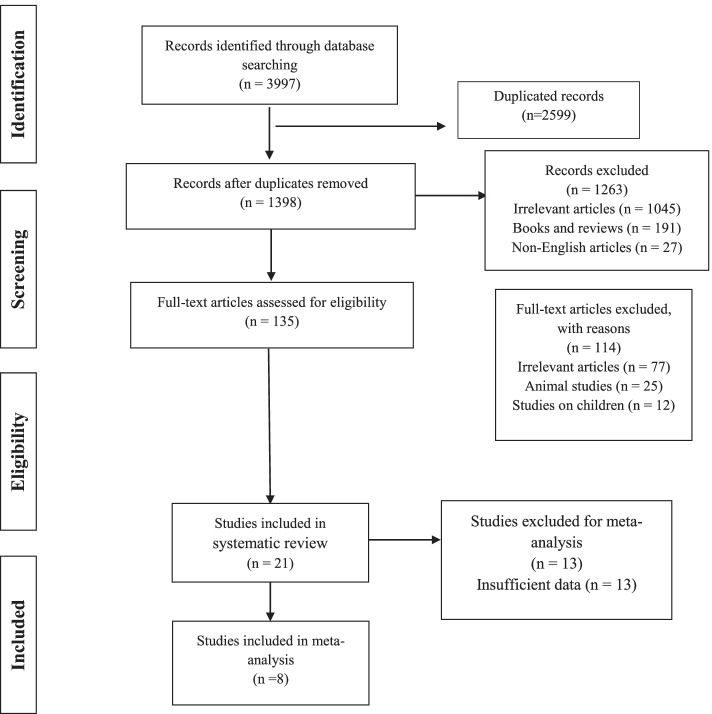
Flowchart of study selection and inclusion of results. Data from 8 papers were included in the meta-analysis

The initial search recognized 3997 articles and 1398 of them remained after excluding duplicates. After screening the title and abstracts, 1332 articles were excluded, and 66 articles remained for further assessment. The full texts of remaining studies were reviewed carefully by two researchers. Any discrepancy was resolved by the third reviewer. Finally, twenty-one articles were included in the systematic review [7–16, 21–31], and eight of them were included in the meta-analysis [8, 9, 12, 13, 15, 16, 28, 29] (Fig. 1). There were seven cohort, seven case-control, and seven cross-sectional studies. The number of participants varied from 45 to 13,535. Table 1 show the characteristics of all included studies.

**Table 1:** Description and summary of data from 21 studies that investigated associations between DNA methylation and cardiometabolic risk factors

First author	Country/year	Study Type	Sample size	Study Characteristic	Tissue	Gene Site	Adjusted covariates	Results	Quality assessment^*2^
Daniel Castellano-Castillo [9]	Spain 2018	case-control	108	Non MetS (55) Age:48.4 ± 13.9 BMI:29.8 ± 7.9 M/F: 52/48 MetS (53) Age:52.7 ± 14.6 BMI:36.4 ± 10.9 M/F: 44/56	Visceral Adipose Tissue	LINE-1 P1-P6^*1^	age, sex	Negative correlation between LINE-1 P2 and the MetS index and no correlation at P1, P3, P4, P5, P6. Negative correlations between LINE-1 P1, P2 and P5 and glucose levels. No correlation between LINE-1 and P3, P4, P6 and glucose levels.	18
Valérie Turcot [7]	Canada 2012	case-control	176	severely obese undergoing a biliopancreatic diversion with sleeve gastrectomy to treat obesity Non MetS (98) Age mean: 34.9 ± 8.1 BMI mean:49.8 ± 8.4 M/F: 14/84 MetS (88) Age mean: 35.3 ± 7.3 BMI mean:53.8 ± 10.8 M/F: 20/68	Visceral Adipose Tissue	LINE-1	age, sex and smoking	LINE-1%meth levels in VAT were associated negatively with fasting plasma glucose, blood pressure and MetS.	18
Jose Luiz Marques-Rocha [10]	Brazil 2016	cross-sectional	156	M/F:91/65 Age mean: 23.1 ± 3.5 BMI mean: 22 ± 2.9	WBC	LINE-1	calories, sex, age, smoking, regular physical activity	LINE-1 methylation associated with body fat, waist girth and waist-to-hip ratio, total fat mass, blood pressure.	20
Mark S Pearce [8]	UK 2012	Cohort (The Newcastle Thousand Families Study)	228	Age: 49–51 y BMI mean: 25.70 (22.94–28.93) M/F: 85/143	peripheral blood samples	LINE-1	Sex	Increased LINE-1 DNA methylation was associated with increasing fasting glucose, total cholesterol, total triglycerides, and LDL cholesterol and with decreasing HDL cholesterol, and HDL:LDL ratio	18
Haley L. Cash [21]	US 2011	case-control	355	American Samoa (198) Age mean: M (57): 36.1 ± 5.4 F(141):29.7 ± 6.8 BMI mean: M (57): 34.8 ± 6.6 F(141): 36.0 ± 9.2 Samoa (157) Age mean: M (31): 39.2 ± 5.7 F(126): 29.2 ± 6.2 BMI mean: M (31): 28.7 ± 5.4 F(126): 31.1 ± 5.8	peripheral lymphocyte	LINE-1	age, sex, BMI	Significant positive association between BMI and HDL with LINE-1 methylation Significant negative association between LDL and LINE-1 methylation	17
Stacey E Alexeeff [22]	US 2013	longitudinal study (cohort)	798	M/F: Age mean: 74 (55–100 y) BMI mean: 27.5	buffy coat	LINE-1	BMI, age, smoking, T2D, alcohol, race, IHD/MI, Neut count, season, day of week.	LINE-1 methylation Inversely associated with DBP, LINE-1 methylation association with SBP was weak.	17
Yoshiki Tsuboi [13]	Japan 2018	cross-sectional study	420	M/F:187/233 Age mean: 61.46 y BMI mean: 24.16	WBC	LINE-1	sex, age, smoking, alcohol, BMI, CRP, anti hyperlipidemic drug use	Significant positive association between LINE-1 DNA methylation and LDL/HDL ratio. Negative and weak association between LINE-1 DNA methylation and HDL.	19
Carolina Ferreira Nicoletti [14]	Brazil-Spine 2016	cross-sectional study	45	control group (9) normal weight individuals M/F: 0/9 Age mean: 31.7 ± 8.6 BMI mean: 22.0 ± 2.0 obese with energy restriction group (22) M/F: 0/22 Age mean: 52.6 ± 9.9 BMI mean: 38.2 ± 3.7 obese with bariatric surgery group (14) M/F: 0/14 Age mean: 35.5 ± 10.1 BMI mean: 44.6 ± 6.2	buffy coats	LINE-1	age and BMI	Significant association between LINE-1 methylation and serum glucose levels.	18
Liliane Pfeiffer [11]	Germany 2014	Augsburg cohort	2747	M/F: 1341/1406 Age mean: 61.63 y BMI mean: 27.46	whole blood samples	ABCG1 cg06500161 SREBF1 cg11024682	age,sex, BMI, smoking, alcohol, lipid lowering drugs, physical activity, history of MI, hypertension, HbA1c, CRP, WBC count	Opposite directions ABCG1 methylation association with HDL and triglyceride levels. Association between triglyceride levels and ABCG1, SREBF1.	20
Alexis C. Frazier-Wood [23]	US 2014	GOLDN cohort	994	Discovery (663) M/F: 312/351 Age mean: 48.6 ± 16.4 BMI mean: Replication (331) M/F: 165/166 Age mean: 47.7 ± 16.2 BMI mean:	CD4 + T cells	ABCG1 cg06500161	age, sex, study site, and the first four principal components generated to estimate T-cell purity as fixed effects, and pedigree as a random effect using the lmekin function of the kinship package in R	LDL associated with ABCG1 methylation.	18
Tasnim Dayeh [12]	Sweden 2016	Botnia prospective study	258	non-diabetic at baseline: Controls (129) M/F:62/67 Age mean: 51.4 ± 9.1 BMI mean: 27.6 ± 3.0 Converters(129) M/F:65/64 Age mean: 52.8 ± 12.3 BMI mean: 28.8 ± 4.3	blood	ABCG1 cg06500161 PHOSPHO1 cg02650017	age, gender, fasting glucose, and family relation	Positive correlation between DNA methylation at the ABCG1 locus cg06500161 with BMI, HbA1c, fasting insulin, and triglyceride levels. Positive correlation between DNA methylation at the PHOSPHO1 locus cg02650017 with HDL levels. DNA methylation at the ABCG1 locus cg06500161: 9% increased risk for future T2D DNA methylation at the PHOSPHO1 locus cg02650017: 15% decreased risk for future T2D	19
Eliza Walaszczyk [15]	Netherland 2017	case–control sample Lifelines cohort	198	Type 2 diabetic (100) M/F: 52/48 Age mean: 62 (53–69y) BMI mean: 30.8 ± 4.7 Control individuals (98) M/F: 44/54 Age mean: 50 (46–63y) BMI mean: 25.3 ± 3.6	whole blood	ABCG1 SREBF1	age, sex, measured blood cell composition, plate number and position on the plate as covariates	ABCG1 methylation associated with FBS, TG and TC SREBF1 methylation associated with FBS, TG, TC and LDL	18
John C Chambers [16]	London 2015	prospective nested case-control (LOLIPOP)	13,535	M/F: 8175/5360 Age mean: 49.1 ± 10.9 BMI mean: 27.0 ± 4.4	peripheral blood leucocytes	SREBF1 PHOSPHO1 ABCG1	Age, sex	Methylation at SREBF1*,* PHOSPHO1, and ABCG1association with quantitative measures of total and regional body fat distribution	18
Jennifer Kriebel [25]	Germany 2016	KORA F4 Study	1448	non-diabetic individuals M/F: 682/766 Age mean: 59 (32-81y) BMI mean: 27.1	whole blood	SREBF1 cg11024682 ABCG1 cg06500161	age, sex, estimated white blood cell proportions, smoking, BMI	Significant associations between cg06500161 (ABCG1) methylation and waist circumference, triglycerides, fasting glucose, and 2-hour glucose, fasting insulin, CD8 + T cells, and monocytes Significant associations between cg09694782 (SREBF1) methylation and age, fasting insulin, and HOMA-IR.	19
Kim V. E. Braun [24]	Netherland 2017	Rotterdam Study	1485	Discovery (725) M/F: 336/389 Age mean: 59.9 ± 8.2 BMI mean: 27.6 ± 4.6 Replication (760) M/F: 334/426 Age mean: 67.7 ± 5.9 BMI mean: 27.8 ± 4.2	whole blood	SREBF1 cg11024682 ABCG1 cg06500161	age, gender, current smoking, leukocyte proportions, array number, and position on array	Association between ABCG1 methylation and HDL Association between ABCG1, and SREBF1 methylation with triglycerides	21
Ping Peng [26]	China 2014	case-control	139	CHD patients (85) M/F: 58/67.4 Age mean: 61.33 ± 9.22 control group (54) M/F: 31/57.4 Age mean: 56.35 ± 9.0	peripheral blood	ABCG1	age, gender, smoking, lipid level, hypertension, and diabetes	Significant statistical association of the promoter Hyper-methylation of the ABCG1 gene with CHD risk ABCG1 and GALNT2 gene promoter regions are positively associated with CHD both in the male group	20
S. Sayols-Baixeras [27]	Spain 2016	REGICOR and Framingham study cross-sectional	2858	REGICOR discovery sample (645) M/F: 316/329 Age mean: 63.2 ± 11.7 BMI mean: 26.9 ± 4.1 Framingham (2542) M/F: 1164/1378 Age mean: 66.3 ± 8.9 BMI mean: 28.2 ± 5.4	whole peripheral blood	SREBF1 PHOSPHO1 ABCG1	sex, age, smoking status,batch effect and estimated cell count	Positive association between SREBF2 methylation and TC, in the same direction as the association between SREBF1 and TG. Significant association between methylation levels of SREBF1 and HDL in the opposite direction to that observed with TG. Direct association between PHOSPHO1 methylation and HDLholesterol levels.	21
Simon-Pierre Guay [28]	Canada 2014	Case-controle study	61	severely obese non-FH (30) BMI > 40 familial hypercholesterolemia (61) M/F:61/0	Whole blood	ADRB3	age, waist circumference, fasting triglyceridemia	Higher ADRB3DNA methylation levels were significantly associated with lower low-density lipoprotein cholesterol levels in FH, and with a lower waist-to-hip ratio and higher blood pressure in severely obese men.	17
Raquel PatríciaAtaíde Lima [31]	Brazil 2019	cross-sectional representative study	265	M/F: 79/186 Age mean: 40.3 ± 14.3 (20–59) BMI mean: 27.08 ± 5.88	leukocytes	ADRB3	–	LDL above the median had a 164% higher chance of ADRB3 hyper-methylation, whereas individuals with triglyceride values above the median had a higher chance of hyper-methylation.	16
Andrée-Anne Houde [29]	Canada 2015	Cross-sectional	73	men and premenopausal women (BMI > 40 kg/m2) undergoing bioliopancreatic diversion with duodenal switch to treat obesity (severely obese) M/F: 33/40	whole blood SAT VAT	LEP	Age, sex and waist circumference	LEP DNA methylation levels in blood cells were negatively associated with body mass index (BMI). Fasting LDL levels positively correlated with DNA methylation levels at LEP-CpG11 and -CpG17 in blood and SAT and with ADIPOQ -CpGE1 and - CpGE3 DNA methylation levels in SAT and CpGE1 in VAT. Associations between LDL levels and both LEP and ADIPOQ DNA methylation levels.	18
Jonathan Y Huang 2017 [30]	Israel	Sub-cohort	589	M/F: 0/589 Age mean:32 BMI mean: 27.08 ± 5.88 maternal pre-pregnancy BMI ≥27 kg/m2 and offspring birth weight ≤ 2500 g or ≥ 4000 g	peripheral blood (buffy coat)	LEP	ethnic origin, offspring age at blood draw, maternal characteristics (pre-pregnancy BMI, gestational weight gain, age, parity, education), paternal characteristics (education and smoking status), offspring variables (childhood overweight, education, parity, current smoking status)	ABCA1 methylation appeared to be directly related to both maternal gestational weight Gain and some markers of glucose homeostasis. LEP methylation associated with waist-to-hip ratio	19

*1: They used a tested assay which included 6 CpG sites (P1–6) for the analysis of LINE-1

*2: Based on STROBE checklist

*Abbreviation*: *M* male, *F* female, *BMI* body mass index, *MetS* metabolic syndrome, *VAT* visceral adipose tissue, *WBC* white blood cells, *FH* familial hypercholesterolemia, *SAT* subcutaneous adipose tissue, *IHD* ischemic heart disease, *MI* myocardial infarction, *DBP* diastolic blood pressure, *SBP* systolic blood pressure

### Association between DNA methylation and HDL levels

**Fig. 2:**
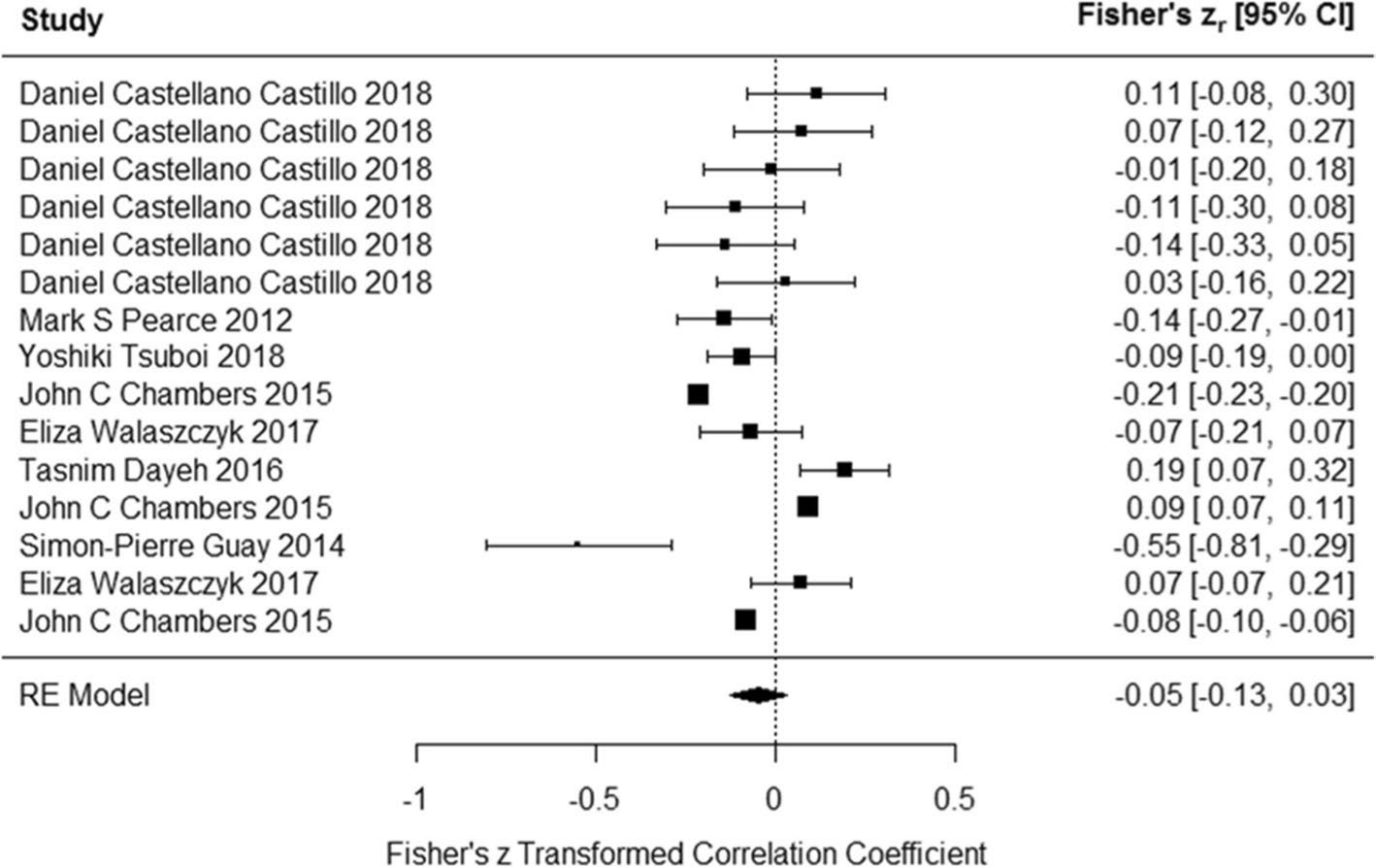
Associations of DNA methylation and HDL levels. DNA methylation had inverse association with serum HDL levels. However, the results were not significant (In Daniel Castellano-Castillo et al. study, methylation of six CpG site of LINE-1 gene in relation to HDL levels were assessed. In Eliza Walaszczyk et al. study, association between methylation of ABCG1 and SREBF1 genes with HDL levels were investigated. In John C Chambers et al. study methylation of ABCG1, PHOSPHO1 and SREBF1 genes in relation to HDL levels were assessed)

Results of meta-analysis on studies indicated that the DNA methylation had inverse association with serum HDL levels (Fisher’s z_r_ = − 0.048 (95% CI:-0.126 to 0.029)) using the random effects model (Fig. 2). However, the results were not significant. Test for heterogeneity (Q = 669.527, *P* < 0.0001) suggested the heterogeneity among the true effects was significant. Funnel plot did not show asymmetry (Z = -0.256, *p* = 0.79).

### Association between DNA methylation and LDL levels

**Fig. 3:**
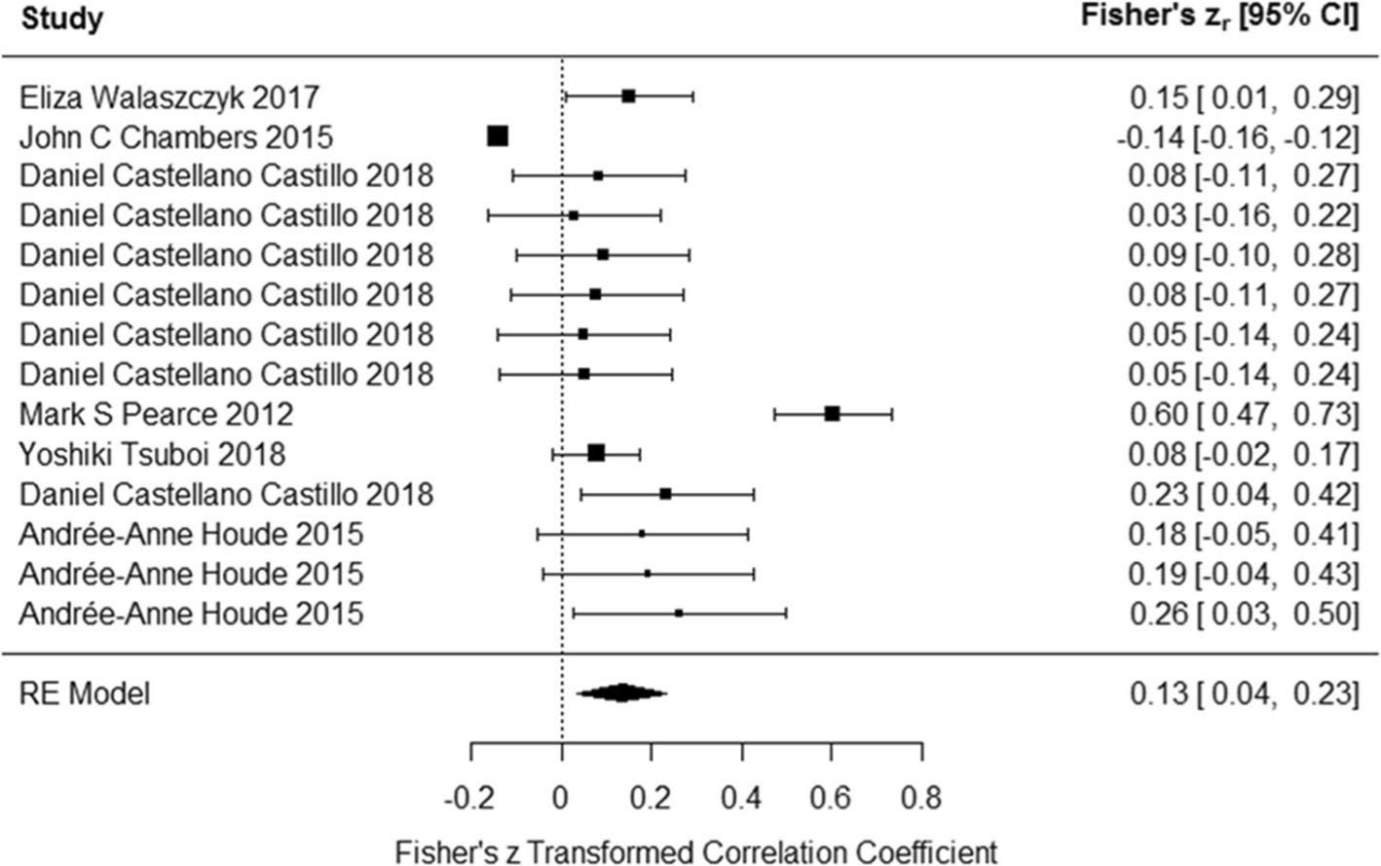
Associations of DNA methylation and LDL levels. DNA methylation had positive association with serum LDL levels. (In Daniel Castellano-Castillo et al. study, methylation of six CpG site of LINE-1 gene in relation to LDL levels were assessed. In Andrée-Anne Houde et al. study, association between methylation of LEP gene and LDL levels in subcutaneous adipose tissue, visceral adipose tissue and whole blood were investigated)

Results of meta-analysis on studies indicated that the DNA methylation had positive association with serum LDL levels (Fisher’s z_r_ = 0.134 (95% CI: 0.035 to 0.233)) using the random effects model (Fig. 3). Test for heterogeneity (Q = 208.05, *P* < 0.0001) suggested the heterogeneity among the true effects was significant. Funnel plot did not show asymmetry (Z = 1.04, *p* = 0.29).

### Association between DNA methylation and HbA1C

**Fig. 4:**
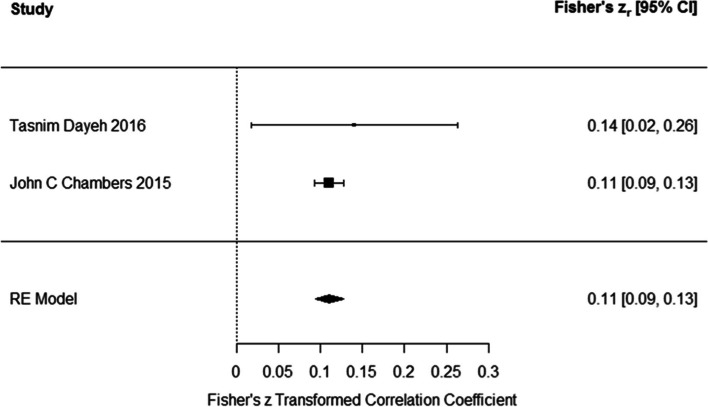
Associations of DNA methylation and HbA1c. DNA methylation had positive association with HbA1C

Results of meta-analysis on two studies indicated that the DNA methylation had positive association with HbA1C (Fisher’s z_r_ = 0.1110 (95% CI: 0.094 to 0.127)) using the random effects model (Fig. 4). Test for heterogeneity (Q = 0.232, *P* = 0.62) suggested the heterogeneity among the true effects was not significant. However, the number of total valid studies was two and there was very low sample size for meta-analysis. Confidence intervals for heterogeneity parameters were very wide.

### Association between DNA methylation and blood glucose

**Fig. 5:**
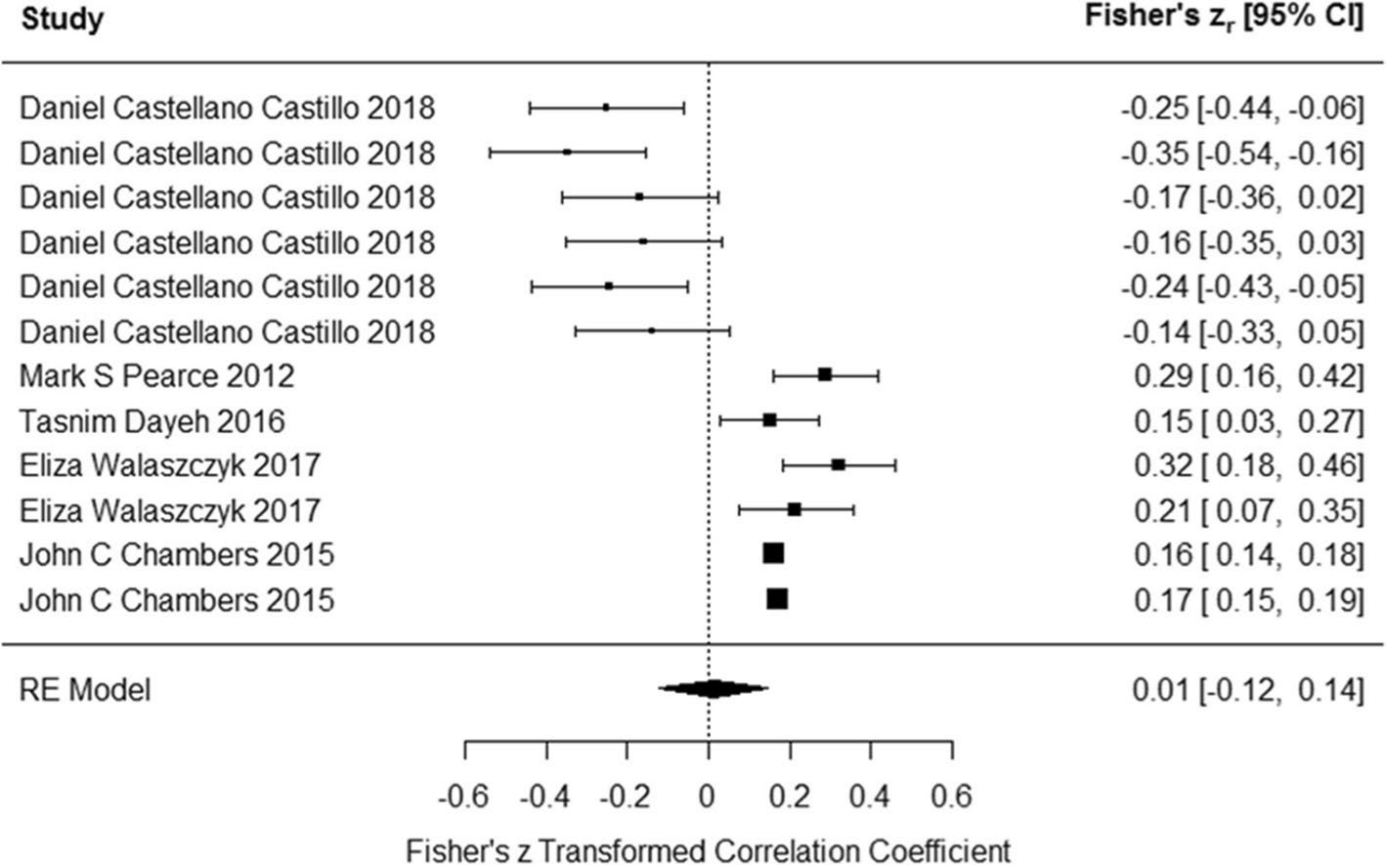
Associations of DNA methylation and blood glucose. DNA methylation had positive association with blood glucose levels. However, the results were not significant. (In Daniel Castellano-Castillo et al. study, methylation of six CpG site of LINE-1 gene in relation to blood glucose levels were assessed. In Eliza Walaszczyk et al. and John C Chambers et al. studies, association between methylation of ABCG1 and SREBF1 genes with blood glucose levels were investigated)

Results of meta-analysis on studies indicated that the DNA methylation had positive association with blood glucose levels (Fisher’s z_r_ = 0.011 (95% CI:-0.121 to 0.144)) using the random-effects model (Fig. 5). However, the results were not significant. Test for heterogeneity (Q = 104.3606, *P* < 0.0001) suggested the heterogeneity among the true effects was significant. Funnel plot shows asymmetry (Z = -2.670, *p* = 0.007). Based on the regression test for funnel plot asymmetry, there was a significant publication bias among the results. So, we need to add two studies with positive correlation coefficient.

### Association between DNA methylation and blood pressure

**Fig. 6:**
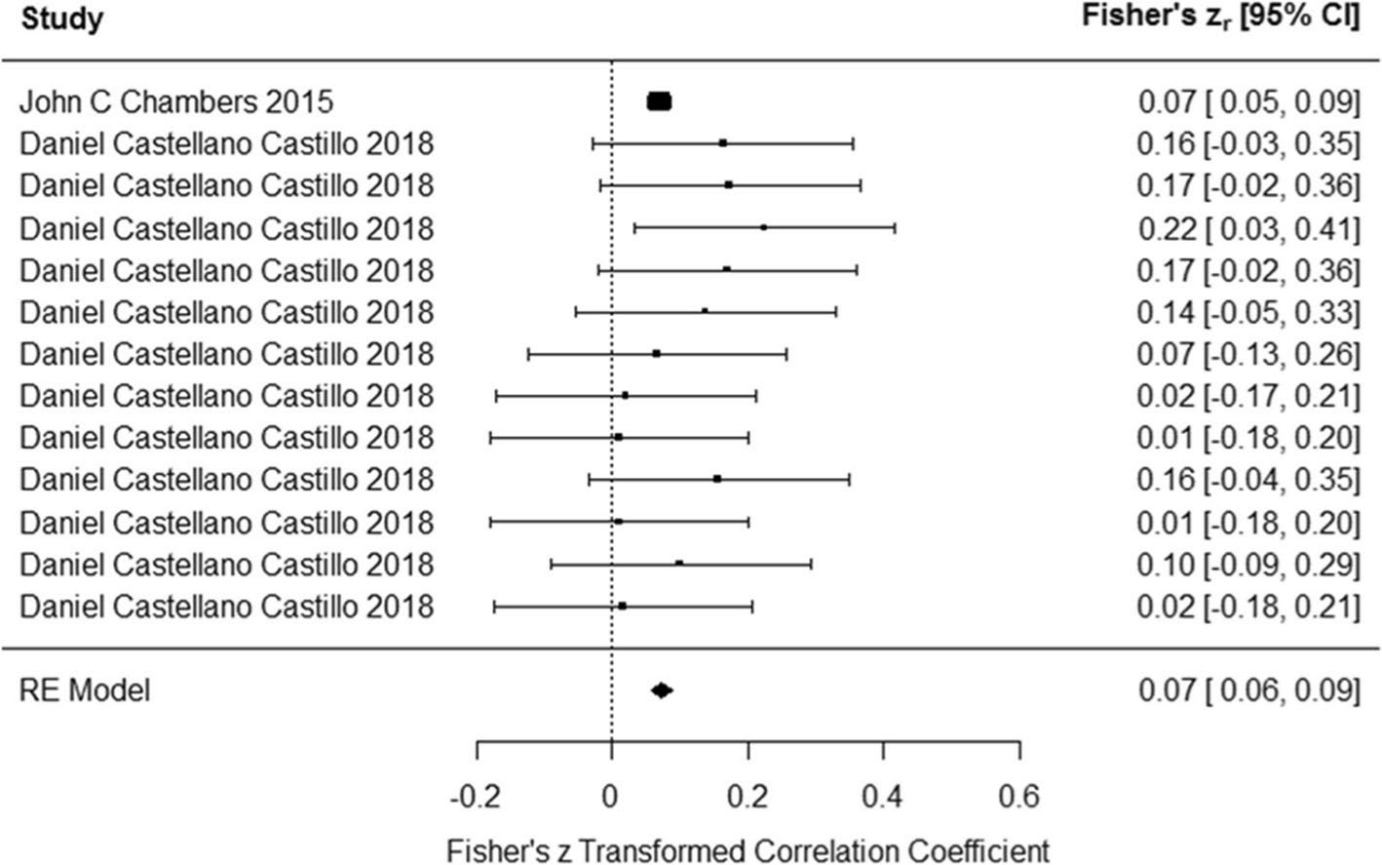
Associations of DNA methylation and blood pressure. DNA methylation had significant positive association with blood pressure (In Daniel Castellano-Castillo et al. study, methylation of six CpG site of LINE-1 gene in relation to systolic blood pressure levels and six CpG site in relation to diastolic blood pressure were assessed)

The findings of this meta-analysis on studies showed that DNA methylation had significant positive association with BP (Fisher’s z_r_ = 0.073 (95% CI: 0.056 to 0.089)) using random effects model (Fig. 6). Test for heterogeneity (Q = 8.087, *P* = 0.77) suggested the heterogeneity among the true effects was negligible. Funnel plot did not show asymmetry (Z = -1.141, *p* = 0.25).

The main reason of heterogeneity in our study was the difference in the gene sequences and sites of methylation in researches. Study participant characteristics such as age, gender and medical history, differences in study design and variation in results were reasons of heterogeneity.

## Discussion

The current systematic review and meta-analysis showed the association between DNA methylation of LINE1, ABCG1, PHOSPHO1, SREBF1, LPL, ADRB3 genes and cardiometabolic risk factors, including HDL, LDL, blood glucose, blood pressure, and HbA1C.

### DNA methylation and levels of HDL and LDL

Our findings did not show any significant association between DNA methylation of LINE1, ABCG1, SREBF1, PHOSPHO1 and ADRB3 genes and HDL levels. However, we found a positive association between DNA methylation of LINE1, ABCG1 and LEP genes and LDL levels. The majority of the included studies confirmed the association between DNA methylation of the mentioned genes and the lipid profile [12, 15, 16, 24, 25].

The prospective nested case-control study, with 15,353 Indian Asian participants, suggested DNA methylation of PHOSPHO1 and ABCG1 genes were significantly associated with HDL and LDL levels. Also, they showed that the SREBF1 methylation was associated with LDL levels [16]. A study on 228 individuals, aged 49–51 years, showed DNA methylation of the LINE-1 gene was positively associated with total cholesterol, triglycerides, and LDL and negatively associated with HDL and HDL/LDL ratio [8]. Moreover, an investigation on 420 Japanese population showed a positive association between DNA methylation of LINE-1 gene and LDL/HDL ratio and weakly and negatively association with HDL [13].

LINE-1 gene is retro-transposons that have remained active in the human genome. LINE-1 composes 17% of DNA in human [32]. Retro-transposons are a type of genetic component that can copy and paste themselves into different genomic locations. Therefore, they could be disruptive for nearby genes or regulatory sequences [33]. Epidemiological studies showed LINE-1 methylation was associated with metabolic syndrome and cardiovascular disease [8, 21]. A case-control study on 108 Spanish participants showed no significant association between methylation in six CpG sites of LINE-1 and HDL and LDL levels [9].

Differences in the quantified LINE-1 DNA methylation sites lead to difference in methylation levels [13]. Some studies showed that severe obesity (BMI ≥ 40 kg/m^2^), age and antihyperlipidemic drugs were associated with alterations in LINE-1 DNA methylation and dyslipidemia [13, 29, 34, 35]. Age may be negatively associated with LINE-1 DNA methylation levels [36]. Findings showed that inflammation markers including C-reactive protein (CRP) and interleukin-6 (IL-6) were associated positively with DNA methylation in leukocytes. IL-6 regulates the methyltransferase gene and might lead to epigenetic changes. Obesity and inflammation may fluctuate global DNA hyper-methylation [37, 38].

Some studies showed that women had significantly lower levels of LINE-1 methylation [10, 39, 40]. Different levels of hormonal factors and dietary folate or other one-carbon nutrients in men and women might lead to these differences between genders [41–43]. However, the reason of difference between genders has not been determined exactly and some studies claimed that male/female hormone differences were not the cause of these differences [43, 44].

Physical activity is another factor that is associated with DNA methylation. Higher physical activity levels are associated with higher LINE-1 methylation [45, 46]. Study on subjects with glucose metabolism disorder showed that physical activity intervention improved impaired glucose metabolism and decreased LINE-1 methylation in blood cells [47].

Some studies showed that smoking status can be correlated with DNA methylation [10, 40]. However, another study did not confirm this finding [48].

About the relationship between age and DNA methylation, several studies did not showed any association between LINE-1 methylation levels and age [43, 48].

ABCG1 is another gene sequence in our study that has a critical role in lipid homeostasis. In macrophage, ABCG1 involves in phospholipid transport and improves the efflux of cellular cholesterol to HDL. Also, it has lipid regulating function in other cells like pancreatic beta cells [49]. Due to its role in lipid regulation, ABCG1 affects lipid levels, obesity and diabetes [50]. Ligand-activated retinoic acid receptor and peroxisome proliferator activated receptor α/γ increase ABCA1 and ABCG1 expression [51, 52]. Findings of a large population-based cohort showed ABCG1 methylation at cg06500161 had a reverse association with HDL and triglyceride levels. The expression of ABCG1 may mediate these association [11]. ABCG1 expression might influence on the association between cg06500161 methylation and lipid profile levels [11]. However, there is not any findings that showed a direct relationship between ABCG1 and triglyceride levels. ABCG1 expression is correlated with genetic variants in the ABCG1 promoter and can influence on lipoprotein lipase bioavailability [53].

Methylation-dependent transcription factor binding mediates the reverse relationship between ABCG1 methylation (cg06500161, cg27243685) and ABCG1 mRNA levels. There is opposite association between HDL-C and triglyceride levels and ABCG1 mRNA levels [54].

LEP encodes leptin, a protein that plays an essential role in regulating appetite, energy homeostasis, and obesity. A prospective birth cohort study showed LEP methylation were related to maternal metabolic status and fetal growth in pregnancy. Also, LEP methylation was associated with infancy and childhood obesity [55]. Moreover, leptin influences on immune system, inflammation, hematopoiesis, angiogenesis, reproduction and bone formation [56, 57]. A study on DNA sample of blood, subcutaneous adipose tissue (SAT) and visceral adipose tissue (VAT) showed that DNA methylation levels at LEP-CpG11 and CpG17 in blood and SAT were positively associated with fasting LDL levels [29]. These two CpGs involved in regulation of LEP gene [58, 59]. Findings showed that responsiveness to a low-calorie diet could be correlated with both TNF and LEP DNA methylation [60].

ADRB3 encodes a protein of beta-adrenergic receptors family. This protein improves lipolysis in adipose tissue and thermogenesis in skeletal muscle. Furthermore, ADRB3 mediates the catecholamine-induced activation of adenylate cyclase and has anti-diabetes and anti-obesity effects [61]. An investigation on 61 familial hypercholesterolemia patients showed higher ADRB3 DNA methylation levels were significantly associated with LDL levels. After adjustment with age, blood lipid profile and ADRB3 gene promoter genotype, ADRB3 DNA methylation levels in obese men were lower than familial hypercholesterolemia men [28]. DNA hypermethylation of gene promoter was correlated with reduction of gene expression. However, there was positive association when DNA methylation influenced on the binding of potential transcription repressors [62–64].

### DNA methylation and blood glucose and HbA1C

The findings of the present meta-analysis showed a positive association between ABCG1 methylation and HbA1C. There was no significant association between DNA methylation of LINE1, ABCG1 and SREBF1 genes and glucose levels. A study on Indian-Asian populations reported that DNA methylation of ABCG1, SREBF1 and PHOSPHO1genes were meaningfully associated with HbA1C and glucose levels [16]. Also, an investigation on 228 individuals, aged 49–51 years, from the Newcastle Thousand Families Study (NTFS) showed significant association between LINE-1 methylation and serum glucose levels [8]. Furthermore, a prospective study in Finland on non-diabetic population showed DNA methylation of SREBF1 and ABCG1 genes were associated with HbA1C, glucose levels and type II diabetes risk [12]. However, research on a population undergoing a biliopancreatic diversion with sleeve gastrectomy to treat obesity showed LINE-1 methylation was negatively associated with fasting glucose levels [7].

A population-based study, on 1448 non-diabetic individuals from the region of Augsburg showed association between ABCG1 methylation at cg06500161 and fasting glucose, 2-hour glucose and fasting insulin. In addition, DNA methylation at cg06500161 was oppositely correlated with its expression [25].

PHOSPHO1 that encodes the bone-specific phosphatase involves in energy metabolism disorders. However, the exact mechanism remains unclear [65]. Moreover, due to its role in arterial wall mineralization, PHOSPHO1, can be considered as a therapeutic target for cardiovascular disorders, obesity and diabetes [66]. An animal study on mice reported that PHOSPHO1 inactivation improved glucose homeostasis, including glucose tolerance and insulin sensitivity [65]. However, there are limited studies about PHOSPHO1 methylation and glycemic index, especially insulin-induced indexes.

SREBF1 gene encodes transcription factors that is correlated with energy homeostasis. This factor promotes glycolysis, lipogenesis, and adipogenesis. SREBP1 is a good candidate gene for obesity and obesity-related metabolic disorders like type II diabetes and dyslipidemia because it is an intracellular cholesterol regulator that is located on the endoplasmic reticulum [67].

### DNA methylation and blood pressure

Our study showed DNA methylation of ABCG1 and LINE1 genes were associated with blood pressure. One study on Indian-Asian population showed positive association between ABCG1 methylation and systolic and diastolic blood pressure [16]. DNA methylation at cg06500161 is associated negatively with ABCG1 expression in blood [11, 16]. A study on 1328 European population showed LINE-1 methylation was inversely associated with diastolic blood pressure. But, this association on systolic blood pressure was weaker than diastolic blood pressure [22]. DNA methylation regulates several biological pathways that are involved in hypertension pathogenesis. The renin-angiotensin-aldosterone system (RAAS) is the best known biologic pathway; DNA methylation on related genes is strongly associated with hypertension incidence [68]. However, focus only on single gene methylation or single regulatory pathways and identify specific mechanism related to hypertension progression are difficult [69]. Moreover, other factors including ageing, obesity, smoking, nutrition, sex and etc. affect hypertension that should be considered [70].

The limitation of the present study is selection of only English language articles. It may lead to publication bias and an overrepresentation of effective interventions. The advantages of the present study are assessment of methylation of sex different genes including LINE1, ABCG1, SREBF1, PHOSPHO1, ADRB3 and LEP and different cardiometabolic risk factors including HDL, LDL, HbA1C, blood glucose and blood pressure.

## Conclusion

DNA methylation can influence on cardiometabolic risk factors. Alteration of DNA methylation at LINE-1, ABCG1, and LEP sequences affected LDL levels. ABCG1 and LINE1 methylation influenced on blood pressure. Moreover, ABCG1 methylation was significantly associated with HbA1C as a preventive factor for glycemic status. So, DNA methylation modification should be considered to develop a new methods for prevention, treatment and follow up of cardio-metabolic risk factors. Further studies can be designed on another factors such as drugs and nutrients that can control DNA methylation.

## Data Availability

All data of the current study are available from the corresponding author on reasonable request.

## Abbreviations

NCD: Non-communicable diseases
DNA: Deoxyribonucleic acid
LINE1: Long interspersed nuclear element-1
ABCG1: ATP-binding cassette sub-family G member 1
SREBF1: Sterol regulatory element binding transcription factor 1
PHOSPHO1: Phosphatase orphan 1
ADRB3: Adrenoceptor Beta 3
LEP: Leptin
CPGs: CPG sites
HDL: High-density lipoprotein cholesterol
LDL: Low-density lipoprotein cholesterol
HbA1C: Hemoglobin A1C
BP: Blood pressure
PRISMA: The guideline of Preferred Reporting Items for Systematic Reviews and Meta-Analyses
FBS: Fasting blood sugar
HOMA-IR: Homeostatic model assessment for insulin resistance
RNA: Ribonucleic acid
STROBE: The Strengthening the Reporting of Observational studies in Epidemiology guidelines
T2D: Type 2 diabetes mellitus
SAT: Subcutaneous adipose tissue
VAT: Visceral adipose tissue
NTFS: Newcastle Thousand Families Study
RAAS: The renin-angiotensin-aldosterone system
